# Supplementary material for: The Systems Biology Research Tool: evolvable open-source software
Source: BMC Syst Biol. 2008 Jun 29;2:55. doi: 10.1186/1752-0509-2-55 (PMC2446383; doi:10.1186/1752-0509-2-55)
Supplement: Additional file 1 — SBRT Archive. An archive of the current version of the Systems Biology Research Tool. [file 1752-0509-2-55-S1.zip › sbrt-1.4.0/doc/developers_guide/api/sbrt/shell/text/BoolRelFormatV1.html]

BoolRelFormatV1


|  |  |  |
| --- | --- | --- |
| **Overview** \| **Package** \| **Class** \| **Use** \| **Tree** \| **Deprecated** \| **Index** \| **Help** | |  |
| **PREV CLASS**   **NEXT CLASS** | **FRAMES**    **NO FRAMES**     **All Classes** |  |
| SUMMARY: NESTED \| FIELD \| CONSTR \| METHOD | DETAIL: FIELD \| CONSTR \| METHOD |  |


---


## sbrt.shell.text Class BoolRelFormatV1<E>

```
java.lang.Object
  sbrt.shell.text.BoolRelFormatV1<E>
```

**Type Parameters:**: `E` - the type of element.

**All Implemented Interfaces:**: Format, Formatter<Or<And<E>>>, Parser<Or<And<E>>>, SimpleFormat<Or<And<E>>>

---

``` public class BoolRelFormatV1<E> extends java.lang.Object implements SimpleFormat<Or<And<E>>> ```

This interface is used to represent boolean relationship
formats.

**Author:**
:   This interface was written and documented by
    Jeremiah Wright while in the Wagner lab.

---

**Constructor Summary**

`BoolRelFormatV1(SimpleFormat<E> elementFormat)`
  
          Constructs a new boolean relation format object that uses the
provided format for its elements.


| **Method Summary** | |
| --- | --- |
| `java.lang.String` | `format(Or<And<E>> boolRel)`             Returns a formatted string representation of the provided boolean relationship. |
| `static BoolRelFormatV1<java.lang.String>` | `getBasic()`             Returns an `BoolRelFormatV1` that does not format or parse the elements supplied to it. |
| `Formatter<E>` | `getElementFormatter()`             Returns the formatter for the elements of this set format. |
| `Parser<E>` | `getElementParser()`             Returns the parser for the elements of this set format. |
| `Or<And<E>>` | `parse(java.lang.String set)`             Parses the provided string and returns its corresponding collection of elements as a `LinkedHashSet`. |

**Methods inherited from class java.lang.Object**

`clone, equals, finalize, getClass, hashCode, notify, notifyAll, toString, wait, wait, wait`

**Methods inherited from interface sbrt.shell.text.Formatter**

`format`

**Constructor Detail**

### BoolRelFormatV1

```
public BoolRelFormatV1(SimpleFormat<E> elementFormat)
```

:   Constructs a new boolean relation format object that uses the
    provided format for its elements.

    **Parameters:**: `elementFormat` - the format to be used for elements.


**Method Detail**

### getBasic

```
public static BoolRelFormatV1<java.lang.String> getBasic()
```

:   Returns an `BoolRelFormatV1` that does not format or parse the
    elements supplied to it. In other words, it returns an
    `BoolRelFormatV1` with the most basic possible functionality.

    :   **Returns:**: an `BoolRelFormatV1` that does not format or parse the elements supplied to it.

---


### format

```
public java.lang.String format(Or<And<E>> boolRel)
```

:   Returns a formatted string representation of the
    provided boolean relationship.

    :   **Parameters:**: `boolRel` - the boolean relationship to be formatted. **Returns:**: a formatted string representation of the provided set.

---


### parse

```
public Or<And<E>> parse(java.lang.String set)
```

:   Parses the provided string and returns its
    corresponding collection of elements as a
    `LinkedHashSet`.

    :   **Specified by:**: `parse` in interface `Parser<Or<And<E>>>`
    :   **Parameters:**: `set` - the string to be parsed. **Returns:**: the set of elements corresponding to the provided string.

---


### getElementFormatter

```
public Formatter<E> getElementFormatter()
```

:   Returns the formatter for
    the elements of this set format.

    :   **Returns:**: the formatter for the elements of this set format.

---


### getElementParser

```
public Parser<E> getElementParser()
```

:   Returns the parser for
    the elements of this set format.

    :   **Returns:**: the parser for the elements of this set format.


---


|  |  |  |
| --- | --- | --- |
| **Overview** \| **Package** \| **Class** \| **Use** \| **Tree** \| **Deprecated** \| **Index** \| **Help** | |  |
| **PREV CLASS**   **NEXT CLASS** | **FRAMES**    **NO FRAMES**     **All Classes** |  |
| SUMMARY: NESTED \| FIELD \| CONSTR \| METHOD | DETAIL: FIELD \| CONSTR \| METHOD |  |


---
